# Supplementary material for: Regulatory Role of Vacuolar Calcium Transport Proteins in Growth, Calcium Signaling, and Cellulase Production in Trichoderma reesei
Source: J Fungi (Basel). 2024 Dec 11;10(12):853. doi: 10.3390/jof10120853 (PMC11677643; doi:10.3390/jof10120853)
Supplement: Supplementary file 1 [file jof-10-00853-s001.zip › jof-3324625-Supplementary Materials.pdf]

Journal of Fungi

MDPI

Supplementary Materials: The following supporting information can be downloaded at: https://www.mdpi.com/article/10.3390/jof10120853/s1, Figure S1. Utilized plasmids to construct the deletion cassettes. (a) pJET-*pyr4* and (b) pRS426; Figure S2. Strategy used to confirm the mutant strain. Primers to detect the target gene are in green. Primers for detecting cassette insertion in the correct orientation are in black and blue; Figure S3. Confirmation of the mutant strains Δ*tryvc3* and Δ*tryvc4* using RT-qPCR. Amplification and melt peak chart of Δ*tryvc3* (a) and Δ*tryvc4* (b). The reaction used the indicated sample-primer, e.g. P strain was the sample and Act was the primer. P = parental strain. Act = actin, 55 = Δ*tryvc3*, and 56 = Δ*tryvc4*; Figure S4. Confirmation of the mutant strains Δ*trpmc1* and Δ*tryvc1* using RT-qPCR. Amplification and melt peak chart of Δ*trpmc1* (a) and Δ*tryvc1* (b). The reaction used the indicated sample-primer, e.g. P strain was the sample and Act was the primer. P = parental strain. Act = actin, 58 = Δ*trpmc1*, and 74 = Δ*tryvc1*; Figure S5. Confirmation of the deletion strains using conventional PCR. Deletion of *trpmc1* (*58952*), *tryvc1* (*74057*), *tryvc3* (*55731*) and *tryvc4* (*56440*), according to the primers of Figure S2. The positive mutants in each confirmation phase are highlighted in red. (a) ORF detection for *trpmc1*, (b) 5′ orientation detection for *trpmc1*, (c) 3′ orientation detection for *trpmc1*, (d) ORF detection for *tryvc1*, (e) 5′ orientation detection for *tryvc1*, (f) 3' orientation detection for *tryvc1*, (g) ORF detection for *tryvc3*, (h) 5′ orientation detection for *tryvc3*, (i) 3′ orientation detection for *tryvc3*, (j) ORF detection for *tryvc4*, (k) 5′ orientation detection for *tryvc4*, (l) 3′ orientation detection for *tryvc4*. M—DNA molecular size marker (GeneRuler 1 kb Plus DNA Ladder (Thermo Fisher Scientific, Waltham, MA, USA); Q—positive control (DNA of QM6aΔ*tmus53*Δ*pyr4*—reaction with parental strain DNA; H—negative control (ultrapure water reaction); Figure S6. Growth in different carbon sources. Growth of strains QM6aΔ*tmus53*Δ*pyr4* (parental), Δ*trpmc1*, Δ*tryvc1*, Δ*tryvc3*, and Δ*tryvc4* in minimal media in the presence of 26 mM of different carbon sources for (a) 24 h and (b) 48 h, with and without 10 mM $CaCl_2$ supplementation. The values represent the absorbance readings at 750 nm; and Figure S7. Culture of the parental strain in MA medium + xylose with and without calcium supplementation. Table S1. Primers used in this study. The bold regions are the restriction sites, the underlined regions are the homology regions. Table S2. Vacuolar calcium transport proteins characterized in fungi. References [51–54,71–84] are cited in supplementary materials.

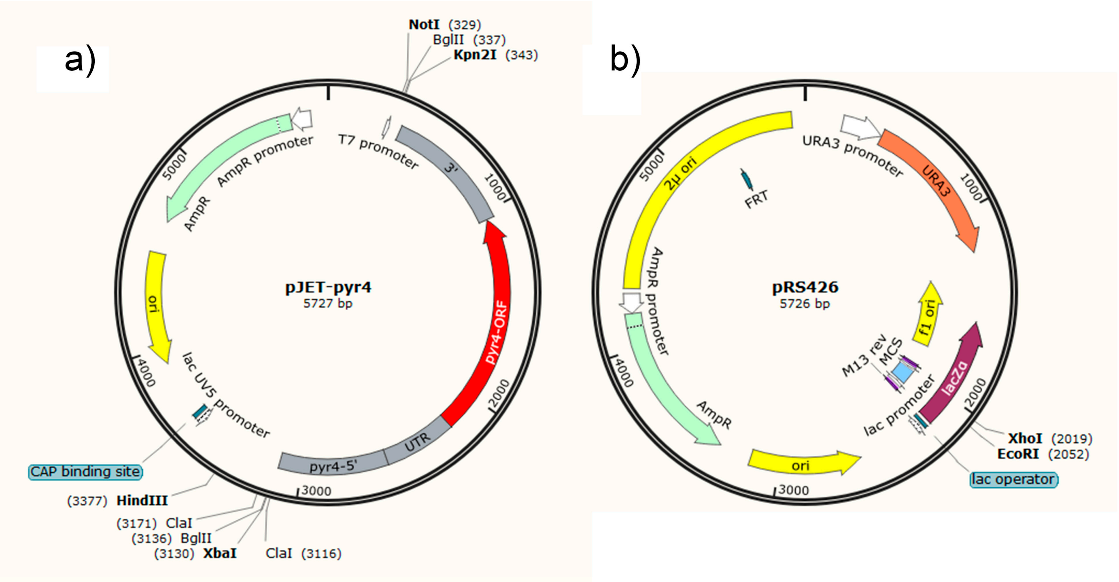

Figure S1. Utilized plasmids to construct the deletion cassettes. a) pJET-*pyr4* [28] and b) pRS426 [31].

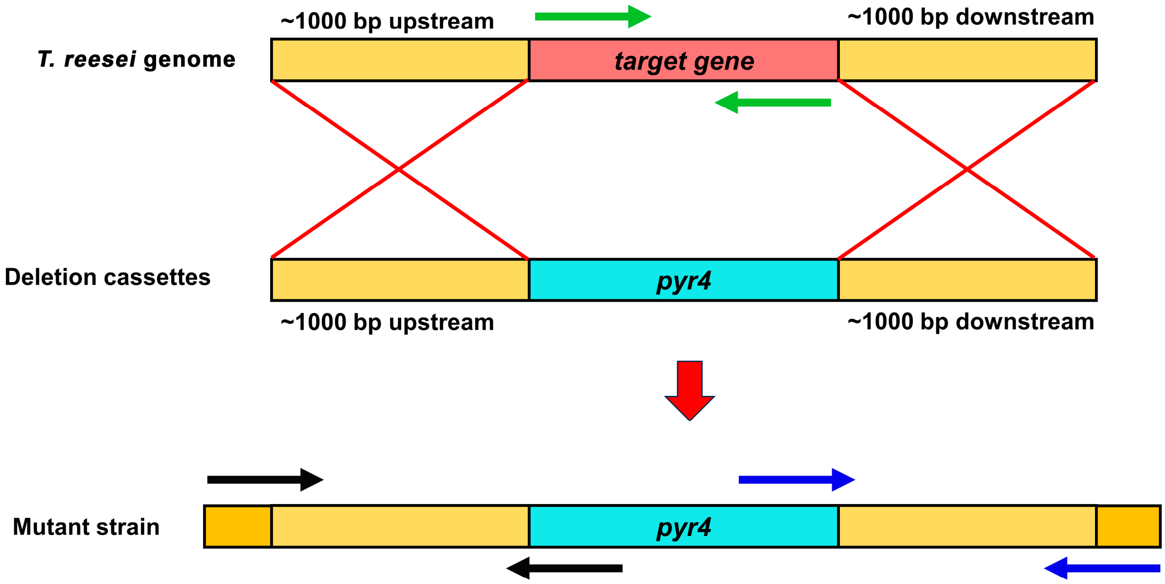

Figure S2. Strategy used to confirm the mutant strain. Primers to detect the target gene are in green. Primers for detecting cassette insertion in the correct orientation are in black and blue.

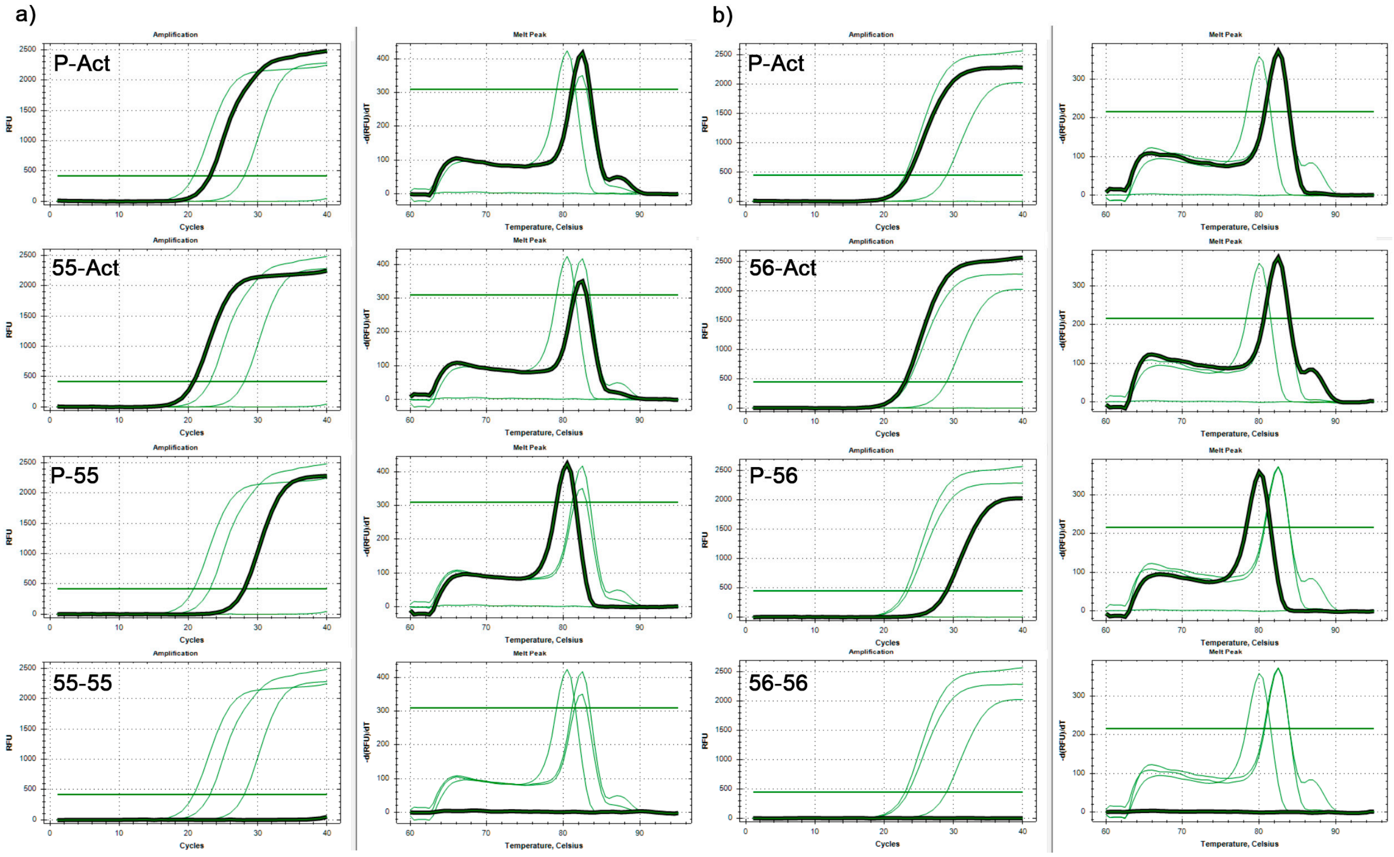

Figure S3. Confirmation of the mutant strains Δ*tryvc3* and Δ*tryvc4* using RT-qPCR. Amplification and melt peak chart of Δ*tryvc3* (a) and Δ*tryvc4* (b). The reaction used the indicated sample-primer, e.g. P strain was the sample and Act was the primer. P = parental strain. Act = actin, 55 = Δ*tryvc3*, and 56 = Δ*tryvc4*.

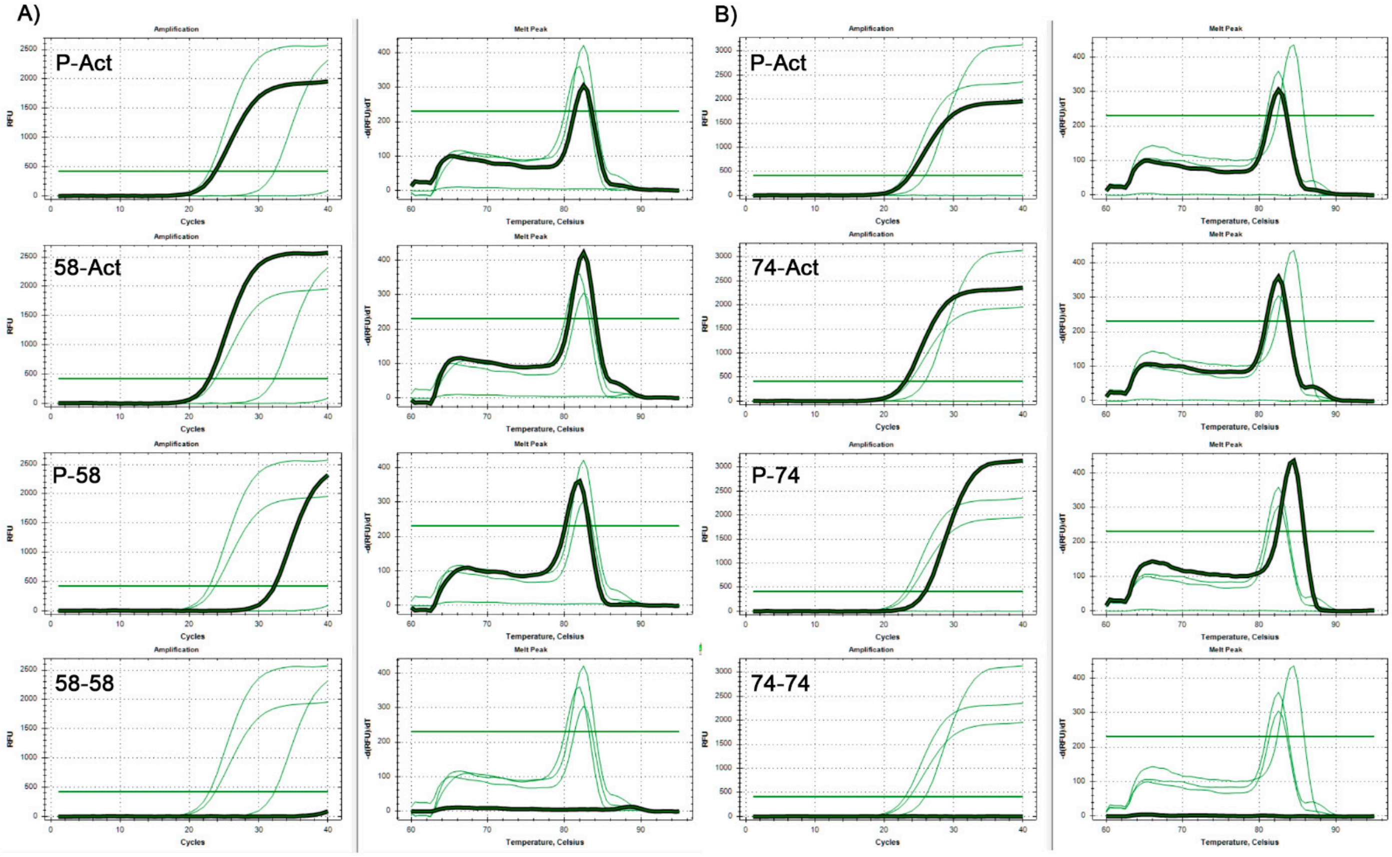

Figure S4. Confirmation of the mutant strains Δ*trpmc1* and Δ*tryvc1* using RT-qPCR. Amplification and melt peak chart of Δ*trpmc1* (a) and Δ*tryvc1* (b). The reaction used the indicated sample-primer, e.g. P strain was the sample and Act was the primer. P = parental strain. Act = actin, 58 = Δ*trpmc1*, and 74 = Δ*tryvc1*.

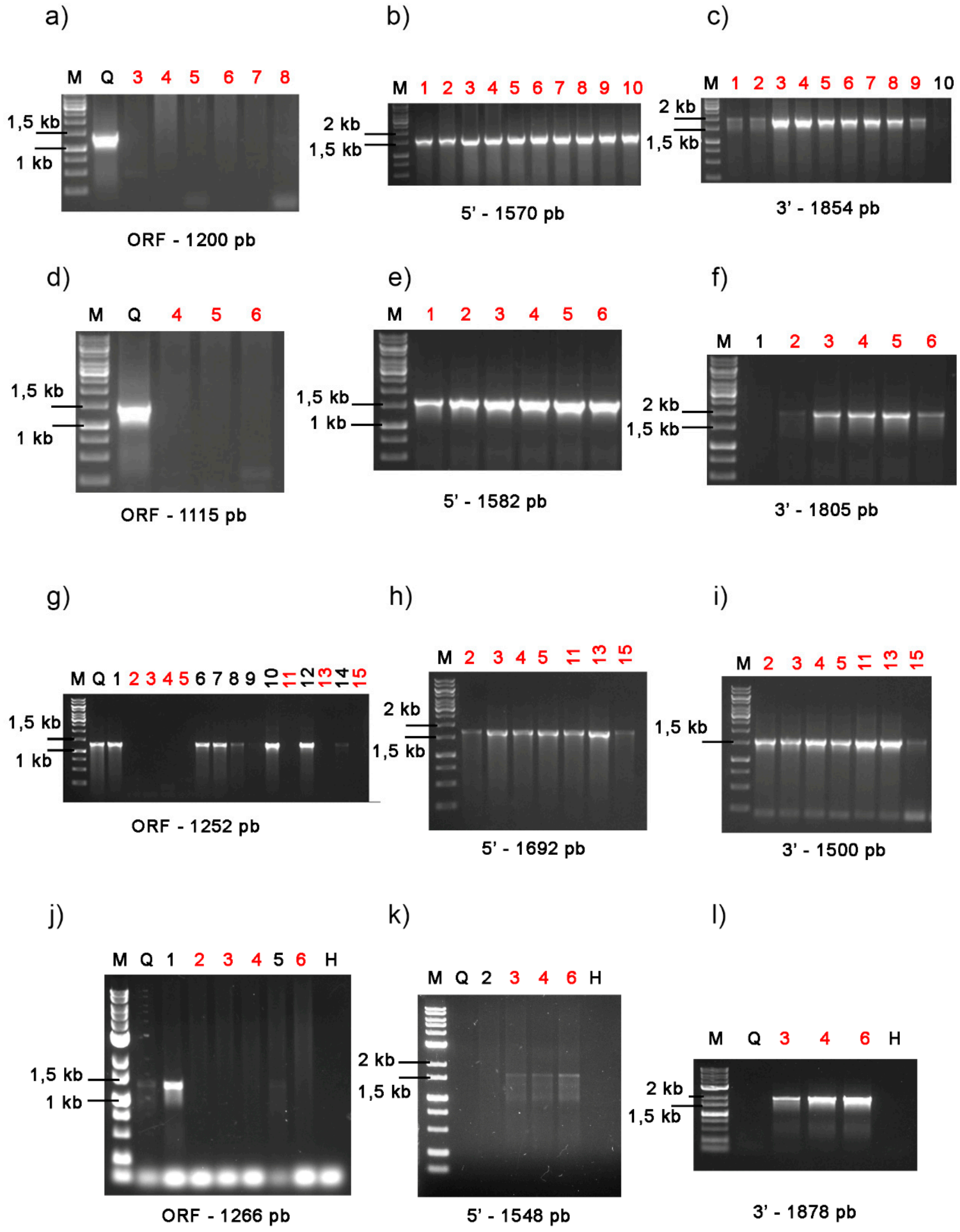

Figure S5. Confirmation of the deletion strains using conventional PCR. Deletion of *trpmc1* (*58952*), *tryvc1* (*74057*), *tryvc3* (*55731*) and *tryvc4* (*56440*), according to the primers of Figure S2. The positive mutants in each confirmation phase are highlighted in red. (a) ORF detection for *trpmc1*, (b) 5′ orientation detection for *trpmc1*, (c) 3′ orientation detection for *trpmc1*, (d) ORF detection for *tryvc1*, (e) 5′ orientation detection for *tryvc1*, (f) 3' orientation detection for *tryvc1*, (g) ORF detection for *tryvc3*, (h) 5′ orientation detection for *tryvc3*, (i) 3′ orientation detection for *tryvc3*, (j) ORF detection for *tryvc4*, (k) 5′ orientation detection for *tryvc4*, (l) 3′ orientation detection for *tryvc4*. M—DNA molecular size marker (GeneRuler 1 kb Plus DNA Ladder (Thermo Fisher Scientific, Waltham, MA, USA); Q—positive control (DNA of QM6aΔ*tmus53*Δ*pyr4*—reaction with parental strain DNA; H—negative control (ultrapure water reaction).

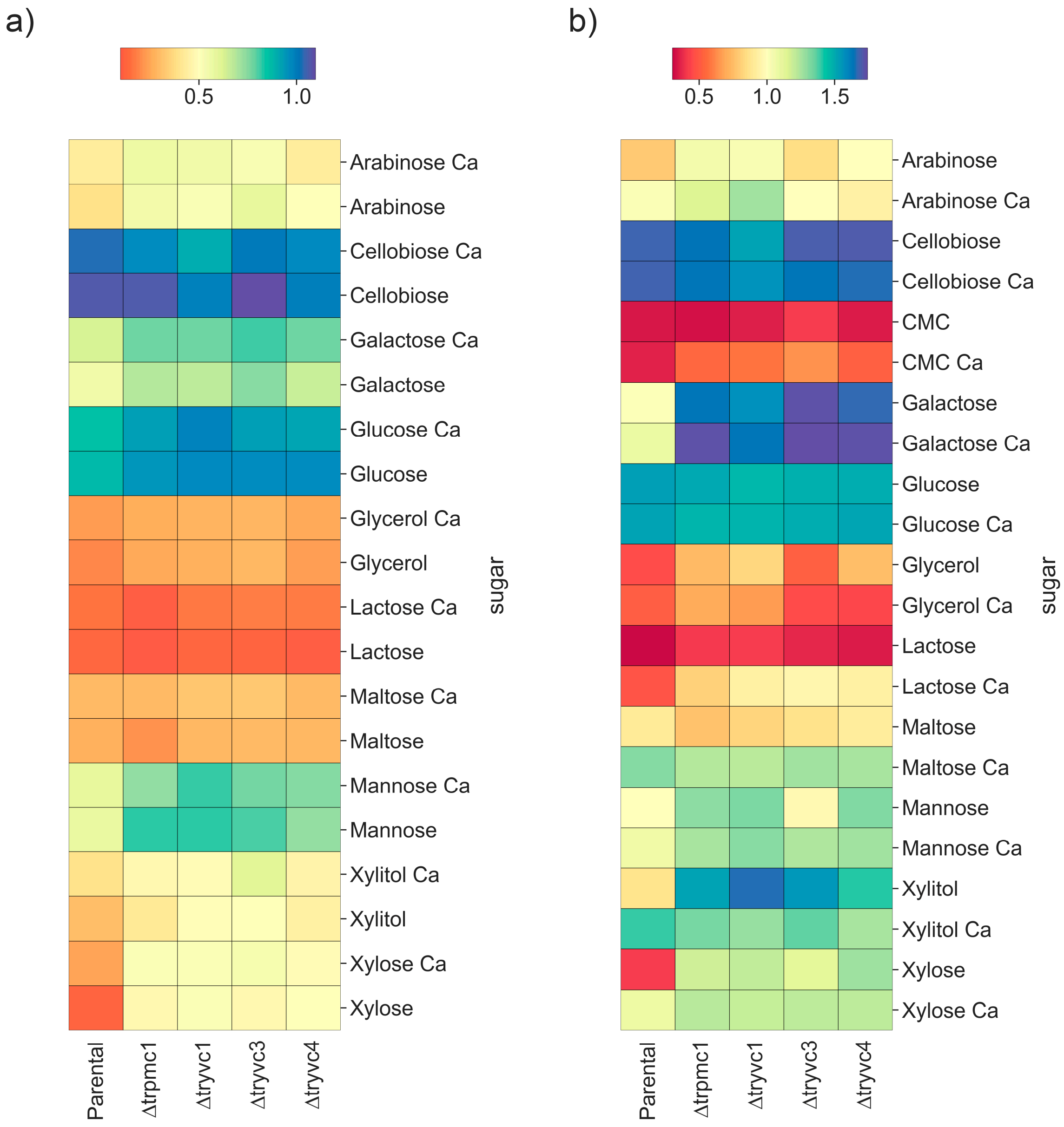

Figure S6. Growth in different carbon sources. Growth of strains QM6aΔ*tmus53*Δ*pyr4* (parental), Δ*trpmc1*, Δ*tryvc1*, Δ*tryvc3*, and Δ*tryvc4* in minimal media in the presence of 26 mM of different carbon sources for (a) 24 h and (b) 48 h, with and without 10 mM $CaCl_2$ supplementation. The values represent the absorbance readings at 750 nm.

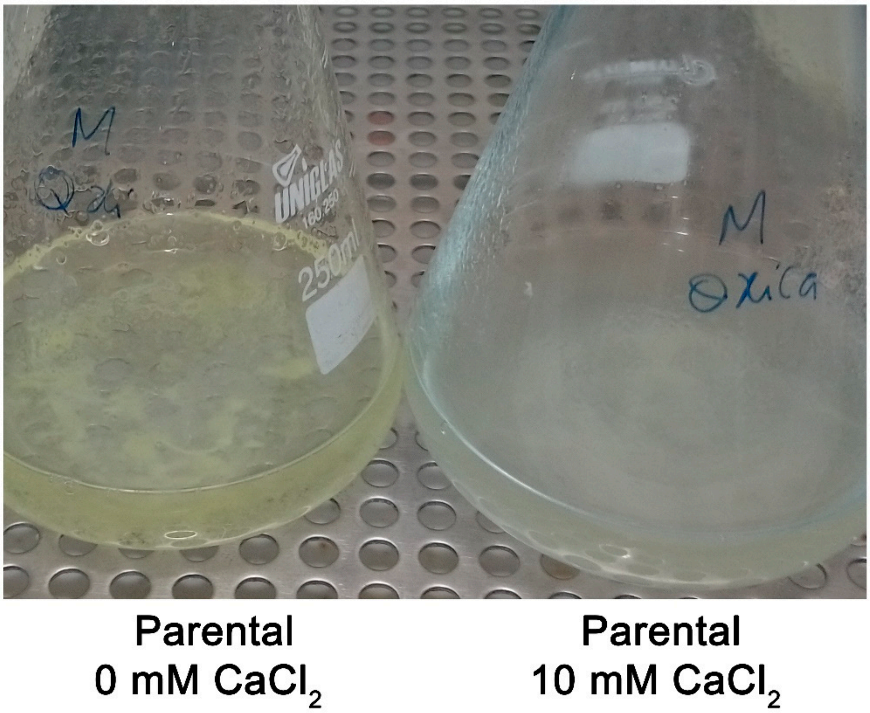

Figure S7. Culture of the parental strain in MA medium + xylose with and without calcium supplementation.

Table S1. Primers used in this study. The bold regions are the restriction sites, the underlined regions are the homology regions.

Primers ligase	Sequence (5′-3′)
*55731*_5F_*Not*I	gggGCGGCCGCTACCTAATCTTGGATCTG
*55731*_5R_*Bgl*II	gggggAGATCTCAAATGTCCGAAGTGGCGTC
*55731*_3F_*Xba*I	gggggTCTAGAGATGGAGCACGAAGGAAAGC
*55731*_3R_*Hind*III	gggggAAGCTTGGACCCTCTGGATTACCAT
56440-5′_F_*Hind*III	gggggAAGCTTGAGTACAACTACTACTGTACA
56440-5′_R_*Cla*I	gggggATCGATGCAATGTAGGGCAGCT
56440-3′_F_*Kpn*2I	ggggTCCGGAAGTAGAGGCGCAGATTAT
56440-3′_R_*Bgl*II	ggggAGATCTGTCCTCGCCAACGCAAA
68169-5′_F_*Hind*III	gggggAAGCTTTCATGTAGGGCTCATCTGCTT
68169-5′_R_*Xba*I	gggTCTAGAGCTGGCGGATTTTGCGAA
68169-3′_F_*Kpn*2I	ggggTCCGGAAGTCTAGCTATCCCATGACAT
68169-3′_R_*Not*I	gggGCGGCCGCAACTCGATCAACTTTAAT
**Primers yeast recombination**	**Sequence (5′-3′)**
*Pyr4*_F_*74057*	TGAGGCTTCGGGGCATCTGCTCTTGCCTTGTCTTGTCTGGGTT
*Pyr4*_R_*74057*	CTGGCTGGTCATTGGAAACTGCGGTTGATTGTTGCCGTCCGTTTC
74057-5F_pRS426	GTAACGCCAGGGTTTTCCCAATCCGACAAGGGGCATCTTGA
74057-5R_*pyr4*	AACCCAGACAAGACAAGGCAAGAGCAGATGCCCCGAAGCCTCA
74057-3F_*pyr4*	GAAACGGACGGCAACAATCAACCGCAGTTTCCAATGACCAGCCAG
74057-3R_ pRS426	GCGGATAACAATTTCACACAGGAAACAGCGTGGGAGCTTAGGGAGACAG
*Pyr4*_F_*58952*	ATTATGGCGAAGAAGGACGACGATCTTGCCTTGTCTTGTCTGGG
*Pyr4*_R_*58952*	TACATGCAGAAGCATTCGTCCGGTTGATTGTTGCCGTCCGTTTC
58952-5F_pRS426	GTAACGCCAGGGTTTTCCCACTCAAGAATGGAGTAGCTATATTG
58952-5R_*pyr4*	CCCAGACAAGACAAGGCAAGATCGTCGTCCTTCTTCGCCATAAT
58952-3F_*pyr4*	GAAACGGACGGCAACAATCAACCGGACGAATGCTTCTGCATGTA
58952-3R_ pRS426	GCGGATAACAATTTCACACAGGAAACAGCGCTCAACTGTGCCATCATCTTCA
**Primers for screening using PCR**	**Sequences (5′-3′)**
*55731*_ORF_F	CCAAGGAGGCCCAGATGTATG
*55731*_ORF_R	GTTGTACGATGGCCATACATCC
*55731*_SC_F	GTGGACTGACAAACGCAA

*55731*_SC_R	CCTCAAGTGCGCAATCTA
*56440*_ORF_F	TCAAGAAGCTGTCCCAGTACATC
*56440*_ORF_R	TCAAGAAGCTGTCCCAGTACATC
*56440*_SC_F	CATCCCTTGAGCATCTGCCT
*56440*_SC_R	AAACTCACCCGCGTCTCTGATA
*58952*_ORF_R	TGGGTAGACGGATAAGGAACTC
*58952*_ORF_F	ATGCTACCCTTACCTACTCTTCCG
*58952*_SC_F	GTGAGACGGTCGGTTACAAGAAG
*58952*_SC_R	ACTTGTATGCTGACCCACCAAG
*68169*_ORF_F	TCATCCCCTTGGGAATAGC
*68169*_ORF_R	CTCCAGATAATCACTTTTGCCG
*68169*_SC_F	CGAATTCCATTGCTGATTGCGC
*68169*_SC_R	CGCTTGCTGTTGGCCACAAAAT
*74057*_ORF_F	TTCGACAGGCGTGGCTATCAAT
*74057*_ORF_R	AGGGCGAAGAATATCACAGAC
*74057*_SC_F	CATGTGCTCTCCCTGATCTGT
*74057*_SC_R	ATCCCAGTCCACGATACGTC
Pyr4-3fwd_WIEN	TGCAACAACACGCGATGG
*Pyr4*_5rev2_WIEN	ACAAGGACTGAGGATGTTCGG
*Pyr4*F_ORF	TGACGGCTTACCTGTTCAAG
*Pyr4*R_ORF	GATGCCAATCAGCTTGTGCG
Primers RT-qPCR	Sequences (5′-3′)
55731-RTF	ACGAATCTACCCTTTGCTGG
55731-RTR	CGTAGTCGGTTGTCCTTCATC
56440-RTF	TGAGTGAATGGATAGATGCCG
56440-RTR	AGAGGGTGCCAATGAGAATC
58952-RTF	CTCTCCAAATGCTACCCTTACC
58952-RTR	CCGACTACATCCGCCATG
68169-RTF	TCTGGTTCAACTTCCTCGC
68169-RTR	CACTCATAGGACCCAACACG
74057-RTF	CGTCATGGGTGTTCTATACTCG
74057-RTR	AATTGCTCCCACTCTTCCAC
*actin*_F	TGAGAGCGGTGGTATCCACG
*actin*_R	GGTACCACCAGACATGACAATGTT

*cam*_F	CCAGATCACCACCAAGGAGT
*cam*_R	AGTCGATGGAGCCGTTGTT
*cna1*_F	CACACTCGACGACCTGAGAA
*cna1*_R	CATTCTGTAACCGGCATCCT
*ace3*_F	GCGTGCTGCAAGATCAGATG
*ace3*_R	TCCAGCCGCGTGAGAATATC
*hac1a*_F	CCTGCAGTGTCAATCGGT
*hac1a*_R	AATGGCCGATGCTGAAAG
*xyl1*_F	GGTTCTACGACGGCAAAAGC
*xyl1*_R	TGTTCAAGTCCATGTCGGCA
*lad1*_F	GATGTCCATTTCTGGCACGC
*lad1*_R	CCCCCAAAAGAGCTGACGAT
*xkl1*_F	GCAAATACAACCTGTGCCCG
*xkl1*_R	GAACGTGATGTCGCTCTTGC
*lxr3*_1	CATACGGCGTCAAGTGCAAG
*lxr3*_R	TTCCACAGCGTCTTGGTCTC
*ypr1*_F	GCTGACTTACCCATCTCTAACC
*ypr1*_R	TGGTTGGATGCTGTTGGAG
*ypr2*_F	TTTCGGCTCCTTCATTCTCG
*ypr2*_R	TTCTGGCACATTCTTGGAGG
*sor1*_F	AGATGCTTCCATTTACTCGGG
*sor1*_R	AATGACAGGAGAAACGGGTG
*xyr1*_F rt	CAATCCTCTCCGTCGCTATTC
*xyr1*_R rt	CTGTTGCCGAATGTGTTGAC
120117cre – F	CTCCTACTCGTCCTTTGTCATG
120117cre - R	GCAAGCATCGTAATGTCGTTG
crz1 F	CCCAAGAGATTCACCAGAGC
crz1 R	TTTCCTGTCATGCTGTCGAG
q*CBHI*for2	CCGAGCTTGGTAGTTACTCTG
q*CBHI*rev2	GGTAGCCTTCTTGACTGAGT
q*CBHII*for	ACAAGAATGCATCGTCTCCG
q*CBHII*rev	TGTTCCACCCGTTGTAGTTG
q*BGLI*for	CTGTACATCACCTACCCATC
q*BGLI*rev	TAGCTGAGATCTCGTCGTC

q*EGL1*for	CCCTCAACACTAGCCACCAG
q*EGL1*rev	AGGTCTTGGAGGTGTCAACG
q*sar1*for	TGGATCGTCAACTGGTTCTACGA
q*sar1*rev	GCATGTGTAGCAACGTGGTCTTT

Table S2. Vacuolar calcium transport proteins characterized in fungi.

Organism	Protein	Access number	Reference
Saccharomyces cerevisiae	YVC1/TRPY1	SGD: S000005613	[71]
	VCX1	SGD: S000002286	[72]
	PMC1	SGD: S000002974	[73]
Candida albicans	YVC1	JGI (SC5314): 58760	[74]
	PMC1	JGI (SC5314): 57421	[75]
Aspergillus fumigatus	YVCA	JGI (Af293 from AspGD): 4483	[76]
	PMCA	NCBI: XP_752453.1	[53]
	PMCB	NCBI: XP_754550.1	
	PMCC	NCBI: XP_746828.1	
Colletotrichum graminicola	CgTRPF1	Ensembl Fungi (M1_001_V1): GLRG_09114	[52]
	CgTRPF2	Ensembl Fungi (M1_001_V1): GLRG_10771	
	CgTRPF3	Ensembl Fungi (M1_001_V1): GLRG_08368	
	CgTRPF4	Ensembl Fungi (M1_001_V1): GLRG_09848	
Fusarium oxysporum	FoYVC1	NCBI: KNB11617.1	[77]
Magnaporthe oryzae	YVC1/Trpy1	Ensembl Fungi (MG8): MGG_09828T0	[78]
	PMC1	Ensembl Fungi (MG8): MGG_02487T0	
	PMC1	Ensembl Fungi (MG8): MGG_04890	
	PMC1	Ensembl Fungi (MG8): MGG_07971T0	
Cryptococcus neoformans	VCX1	NCBI: XP_012046471.1	[79]
	PMC1	NCBI: XP_012049404.1	[80]
Beauveria bassiana	VCX1A	NCBI: XP_008594718.1	[81]
	VCX1B	NCBI: EJP63266.1	
	VCX1C	NCBI: EJP69457.1	
	VCX1D	NCBI: EJP67299.1	
	VCX1E	NCBI: EJP69455.1	
	PMCA	NBCI: EJP70918.1	[54]
	PMCB	NCBI: EJP64689.1	
	PMCC	NCBI: EJP63186.1	
Hansenula polymorpha	PMC1	NCBI: XP_013935643.1	[82]
Schizosaccharomyces pombe	PMC1	NCBI: NP_593890.1	[83]
Aspergillus nidulans	AnPMCA	NCBI: XP_658793.1	[84]
Neurospora crassa	CAX	NCU07075	[51]

Disclaimer/Publisher's Note: The statements, opinions and data contained in all publications are solely those of the individual author(s) and contributor(s) and not of MDPI and/or the editor(s). MDPI and/or the editor(s) disclaim responsibility for any injury to people or property resulting from any ideas, methods, instructions or products referred to in the content.
